# Sex‐Based Differences in Prenatal and Perinatal Predictors of Autism Spectrum Disorder Using Machine Learning With National Health Data

**DOI:** 10.1002/aur.70054

**Published:** 2025-05-19

**Authors:** Ju Sun Heo, Seung‐Woo Yang, Sohee Lee, Kwang‐Sig Lee, Ki Hoon Ahn

**Affiliations:** ^1^ Department of Pediatrics Seoul National University College of Medicine Seoul Korea; ^2^ Department of Pediatrics Seoul National University Children's Hospital Seoul Korea; ^3^ Research Institute of Medical Science Konkuk University School of Medicine Seoul Korea; ^4^ Sanford Consortium for Regenerative Medicine School of Medicine, University of California San Diego California USA; ^5^ AI Center Korea University College of Medicine, Anam Hospital Seoul Korea; ^6^ Department of Statistics Korea University College of Political Science and Economics Seoul Korea; ^7^ Department of Obstetrics and Gynecology Korea University College of Medicine, Anam Hospital Seoul Korea

**Keywords:** autism spectrum disorder, machine learning, risk factors, sex

## Abstract

Autism spectrum disorder (ASD) is a complex neurodevelopmental disorder influenced by genetic, epigenetic, and environmental factors. ASD is characterized by a higher prevalence in males compared to females, highlighting the potential role of sex‐specific risk factors in its development. This study aimed to develop sex‐specific prenatal and perinatal prediction models for ASD using machine learning and a national population database. A retrospective cohort design was employed, utilizing data from the Korea National Health Insurance Service claims database. The study included 75,105 children born as singletons in 2007 and their mothers, with follow‐up data from 2007 to 2021. Twenty prenatal and perinatal risk factors from 2002 to 2007 were analyzed. Random forest models were used to predict ASD, with performance metrics including accuracy and area under the curve (AUC). Random forest variable importance and SHapley Additive exPlanation (SHAP) values were used to identify major predictors and analyze associations. The random forest models achieved high accuracy (0.996) and AUC (0.997) for the total population as well as for the male and female groups. Major predictors included pregestational body mass index (BMI) (0.3679), socioeconomic status (0.2164), maternal age at birth (0.1735), sex (0.0682), and delivery institution (0.0549). SHAP analysis showed that low maternal BMI increased ASD risk in both sexes, while high BMI was associated with greater risk in females. A U‐shaped relationship between socioeconomic status and ASD risk was observed, with increased risk in males from lower socioeconomic backgrounds and females from higher ones. These findings highlight the importance of sex‐specific risk factors, particularly pregestational BMI, and socioeconomic status, in predicting ASD risk.

**Summary**

- This study used national health data and machine learning to develop sex‐specific prenatal and perinatal prediction models for autism spectrum disorder (ASD).
- Major risk factors for ASD were identified as pregestational body mass index, socioeconomic status, maternal age at birth, sex, and delivery institution. Among these, pregestational body mass index and socioeconomic status showed different patterns depending on sex.
- These findings could highlight the importance of considering sex‐specific factors in early prediction and intervention, allowing for more personalized and effective approaches to addressing ASD.

## 1 Introduction

Autism spectrum disorder (ASD) is a neurodevelopmental condition that affects social interaction and communication, leading to early challenges as well as restricted repetitive patterns of behavior, interests, or activities (Lord et al. 2018; Hirota and King 2023). ASD is a spectrum or continuum of severity and functional level. In the Diagnostic and Statistical Manual of Mental Disorders (DSM)‐IV, Asperger's syndrome, childhood disintegrative disorder, pervasive developmental disorder–not otherwise specified, and autistic disorder were separate classifications. However, in the DSM‐V, these classifications have been integrated into ASD. ASD is widely accepted as a complex neurodevelopmental disorder caused by a combination of genetic, epigenetic, and environmental factors (Hirota and King 2023; Halfon and Kuo 2013; Masini et al. 2020; Bhandari et al. 2020). Prenatal and perinatal risk factors are crucial in early brain development and dysregulated epigenetic mechanisms, which can lead to ASD (Bauman and Kemper 2005; O'Reilly et al. 2017; Wilson et al. 2022; Stoccoro et al. 2022).

One of the most striking and consistent findings is the higher prevalence of ASD in males than in females. A meta‐analysis examining the male‐to‐female ratio in ASD found an overall pooled ratio of 4,20 (Loomes et al. 2017). However, when only high‐quality studies were considered, this ratio decreased to 3.32. A recent study analyzing the global prevalence of ASD up to 2021 also reported a median male‐to‐female ratio of 4:2 (Zeidan et al. 2022). This male predominance is likely due to a combination of genetic, hormonal, and environmental factors, including sex‐specific effects of Y‐linked genes, X‐inactivation patterns, parent‐of‐origin allelic imprinting, androgenic hormone exposure, maternal immune activation, and gene–environment interactions (Schaafsma and Pfaff 2014; Werling and Geschwind 2013; McCarthy and Wright 2017). Furthermore, sex‐based differences in ASD are related to differences in gene methylation and maternal risk factors (Stoccoro et al. 2022). Interactions with prenatal or perinatal risk factors may differ based on sex, leading to variations in ASD phenotypes. However, studies are lacking on sex‐specific risk factors or predictors of ASD.

The advent of machine learning techniques provides an unprecedented opportunity to analyze large and complex datasets to uncover intricate patterns and interactions among numerous variables. Recently, machine learning has been widely used to build clinical decision support systems for diagnosing ASD, a challenging disorder due to its highly heterogeneous nature (Wei et al. 2023; Washington et al. 2020). Several studies have developed prediction models for diagnosing and detecting ASD in children based on sociodemographic factors, family characteristics, family medical history, and maternal risk factors (Albahri et al. 2023; Ejlskov et al. 2021; Wei et al. 2024). However, no studies have utilized machine learning models to analyze sex‐based differences in prenatal and perinatal predictors or develop sex‐specific predictive models for ASD.

To address this gap, this study aimed to develop sex‐specific predictive models for ASD using machine learning and a national health insurance database to assess differences in prenatal and perinatal risk factors and their impacts based on sex.

## 2 Methods

### 2.1 Participants

This study utilized population‐based retrospective cohort data from the Korea National Health Insurance (KNHI) claims database. Nearly 97% of the Korean population is enrolled in the KNHI sharing service program, which includes comprehensive information on claims from approximately 50 million Koreans. This extensive dataset captures a wide range of healthcare services and patient demographics, including 75,105 primiparous mothers and their children, all of whom were singleton births in 2007, and included body mass index (BMI) measurements from health examinations. This dataset of 75,105 participants consisted of complete information with pairwise deletion and no missing values. This study was approved by the Institutional Review Board of Korea University Anam Hospital (approval number: 2022AN0184; April 11, 2022), which waived the need for informed consent. The study was performed in accordance with the relevant guidelines and regulations.

### 2.2 Variables

The dependent variable was ASD diagnosed in 2007–2021 (Table S1). The 20 independent variables were categorized as follows: (1) six predictors in 2007, that is, maternal age (years), child's sex (male vs. female), socioeconomic status (SES; measured by insurance fee on a scale from 1 [highest] to 20 [lowest]), medical institution (0 for tertiary general hospital vs. 1 for general hospital, 2 for hospital, and 3 for clinic), preterm birth, and cesarean delivery; (2) five predictors from 2002 (the year the KNHI dataset was established) up to the pre‐pregnancy period, that is, pregestational BMI, pregestational hypertension, pregestational diabetes mellitus (DM), pregestational depression, and pregestational anxiety; (3) six predictors within 10 months before childbirth (no vs. yes), that is, gestational DM, pregnancy‐induced hypertension (PIH), chorioamnionitis, placental abruption, pre‐labor rupture of membranes (PROM), and fetal growth restriction (FGR); (4) one predictor within 12 months before childbirth, that is, antidepressant medication; and (5) two predictors within 12 months of childbirth, that is, postpartum hemorrhage and postpartum depression. These predictors were identified using codes based on the 10th revision of the International Statistical Classification of Diseases and Anatomical Therapeutic Chemical classification codes (Table S1).

### 2.3 Analysis

Logistic regression and random forest models were used to predict the occurrence of ASD. The random forest model was selected due to its superior performance over logistic regression in a related study (Yang et al. 2024). The dataset of 75,105 participants for whom complete information was available was divided into training and validation sets with an 80:20 ratio (60,084 vs. 15,021 cases). The evaluation criteria included accuracy (ratio of correct predictions among the 15,021 cases) and area under the curve (AUC) of sensitivity versus 1–specificity.

Random forest permutation importance was used to identify major predictors of ASD, and SHapley Additive exPlanation (SHAP) values were calculated to analyze the directions of its associations with the predictors. The random forest permutation importance of a predictor reflects the overall decline in accuracy when its data are permuted. The SHAP value of a predictor for a participant indicates the difference in the predicted probability of ASD with versus without the predictor (Yang et al. 2024). For example, if the SHAP values for maternal age range from −0.18 to 0.27 and increase with maternal age, then some participants (children) have SHAP values as low as −0.18 and others as high as 0.27. Including maternal age as a predictor in machine learning will adjust the probability of ASD within this range. However, the maximum value is considered as SHAP values increase with maternal age; this suggests a positive association between maternal age and ASD. The analysis was conducted using R‐Studio 1.3.959 (R‐Studio Inc., Boston, Massachusetts, USA) from January 1, 2024, to June 30, 2024.

## 3 Results

The descriptive statistics and baseline characteristics of the participants are presented in Table 1. Among the 75,105 children born in 2007, 777 (10.3 per 1000 births) were diagnosed with ASD by 2021. Within the ASD group, the male‐to‐female ratio was 3.4 (males: 77.3%; females: 22.7%). The prevalence of ASD was 3.3 times higher among males (601 cases; 15.8 per 1000 male births) than females (176 cases; 4.8 per 1000 female births). The ASD group exhibited a higher prevalence of pregestational depression and hypertension. Additionally, prenatal or postnatal complications, such as gestational DM, PIH, placental abruption, PROM, FGR, preterm birth, and postpartum depression, were also more frequent in the ASD versus non‐ASD group. These patterns were consistent across both male and female subgroups, except for a lower incidence of maternal pregestational depression among females with ASD.

**TABLE 1:** Patients' demographics and baseline characteristics.

(a) Total (*n* = 75,105)			
Variable	ASD (*n* = 777)	Non‐ASD (*n* = 74,328)	*p*
Sex			< 0.001
Male	601 (77.3)	37,537 (50.5)	
Female	176 (22.7)	36,791 (49.5)	
Maternal age	30.0 ± 3.1	29.4 ± 3.1	< 0.001
SES	10.7 ± 4.9	10.3 ± 4.6	0.031
Pregestational BMI	20.9 ± 2.7	20.9 ± 2.7	0.594
Institution			< 0.001
Tertiary general hospital	96 (12.4)	3818 (5.1)	
General hospital	133 (17.1)	8854 (11.9)	
Hospital	279 (35.9)	27,759 (37.3)	
Clinic	269 (34.6)	33,897 (45.6)	
Pregestational anxiety	39 (5.0)	3771 (5.1)	> 0.999
Pregestational depression	26 (3.3)	2159 (2.9)	0.538
Pregestational DM	18 (2.3)	1875 (2.5)	0.796
Pregestational hypertension	17 (2.2)	1195 (1.6)	0.269
Gestational DM	66 (8.5)	5022 (6.8)	0.068
PIH	32 (4.1)	2222 (3.0)	0.089
Chorioamnionitis	4 (0.5)	192 (0.3)	0.297
Placenta abruptio	162 (20.8)	12,130 (16.3)	0.001
PROM	157 (20.2)	11,868 (16.0)	0.002
FGR	176 (22.7)	12,564 (16.9)	< 0.001
Preterm birth	155 (19.9)	11,719 (15.8)	0.002
Cesarean delivery	242 (31.1)	20,313 (27.3)	0.022
Postpartum hemorrhage	8 (1.0)	824 (1.1)	0.962
Postpartum depression	15 (1.9)	709 (1.0)	0.009
Antidepressant	67 (8.6)	6077 (8.2)	0.709

(b) Male (*n* = 38,138)			
Variable	ASD (*n* = 601)	Non‐ASD (*n* = 37,537)	*p*
Maternal age	30.0 ± 3.1	29.4 ± 3.0	< 0.001
SES	10.7 ± 4.8	10.3 ± 4.6	0.012
Pregestational BMI	20.8 ± 2.6	20.9 ± 2.7	0.260
Institution			< 0.001
Tertiary general hospital	68 (11.3)	1982 (5.3)	
General hospital	94 (15.6)	4382 (11.7)	
Hospital	224 (37.3)	14,097 (37.6)	
Clinic	215 (35.8)	17,076 (45.5)	
Pregestational anxiety	34 (5.7)	1943 (5.2)	0.668
Pregestational depression	22 (3.7)	1109 (3.0)	0.375
Pregestational DM	16 (2.7)	990 (2.6)	> 0.999
Pregestational hypertension	12 (2.0)	609 (1.6)	0.601
Gestational DM	50 (8.3)	2568 (6.8)	0.190
PIH	21 (3.5)	1071 (2.9)	0.432
Chorioamnionitis	3 (0.5)	95 (0.3)	0.438
Placenta abruptio	125 (20.8)	6146 (16.4)	0.005
PROM	121 (20.1)	6012 (16.0)	0.008
FGR	133 (22.1)	6320 (16.8)	0.001
Preterm birth	120 (20.0)	5941 (15.8)	0.007
Cesarean delivery	187 (31.1)	10,698 (28.5)	0.183
Postpartum hemorrhage	7 (1.2)	405 (1.1)	> 0.999
Postpartum depression	11 (1.8)	365 (1.0)	0.057
Antidepressant	50 (8.3)	3161 (8.4)	0.980

(c) Female (*n* = 36,967)			
Variable	ASD (*n* = 176)	Non‐ASD (*n* = 36,791)	*p*
Maternal age	30.0 ± 3.2	29.4 ± 3.1	0.024
SES	10.3 ± 5.1	10.3 ± 4.5	0.952
Pregestational BMI	21.1 ± 2.9	20.9 ± 2.7	0.371
Institution			< 0.001
Tertiary hospital	28 (15.9)	1836 (5.0)	
General hospital	39 (22.2)	4472 (12.2)	
Hospital	55 (31.3)	13,662 (37.1)	
Clinic	54 (30.7)	16,821 (45.7)	
Pregestational anxiety	5 (2.8)	1828 (5.0)	0.260
Pregestational depression	4 (2.3)	1050 (2.9)	0.813
Pregestational DM	2 (1.1)	885 (2.4)	0.393
Pregestational hypertension	5 (2.8)	586 (1.6)	0.314
Gestational DM	16 (9.1)	2454 (6.7)	0.258
PIH	11 (6.2)	1151 (3.1)	0.033
Chorioamnionitis	1 (0.6)	97 (0.3)	0.960
Placenta abruptio	37 (21.0)	5984 (16.3)	0.108
PROM	36 (20.5)	5856 (15.9)	0.123
FGR	43 (24.4)	6244 (17.0)	0.011
Preterm birth	35 (19.9)	5778 (15.7)	0.156
Cesarean delivery	55 (31.2)	9615 (26.1)	0.150
Postpartum hemorrhage	1 (0.6)	419 (1.1)	0.721
Postpartum depression	4 (2.3)	344 (0.9)	0.148
Antidepressant	17 (9.7)	2916 (7.9)	0.481

*Note:* The values are presented as mean ± SD for continuous variables and as *n* (%) for categorical variables.

Abbreviations: ASD, autism spectrum disorder; BMI, body mass index; DM, diabetes mellitus; FGR, fetal growth restriction; PIH, pregnancy‐induced hypertension; PROM, pre‐labor rupture of membranes; SES, socioeconomic status.

**FIGURE 1:**
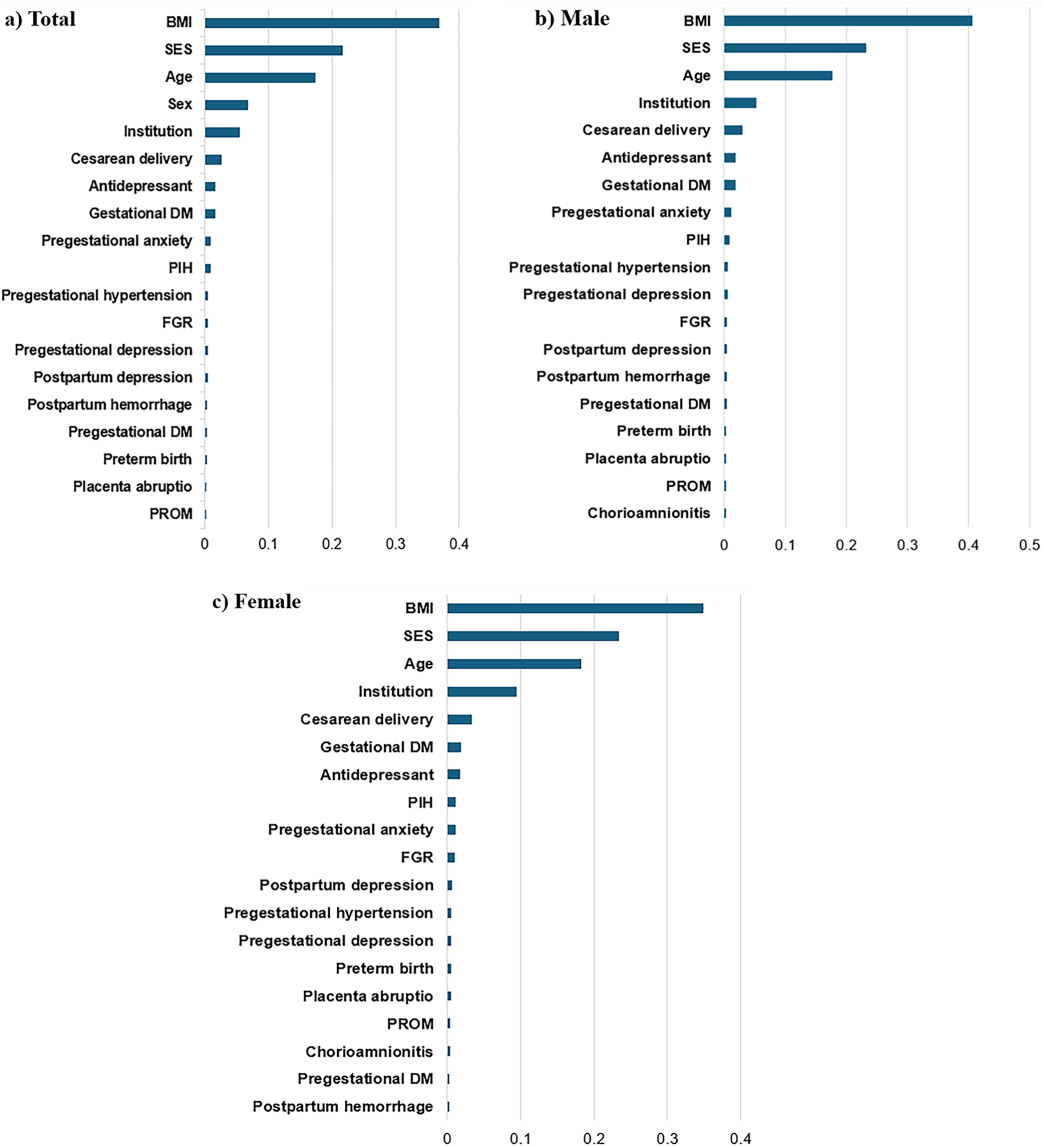
Random forest variable importance for autism spectrum disorder. Random forest variable importance calculates node impurity (Gini) decrease from the creation of a branch on a certain predictor. It is an average over all trees in a random forest with the range of 0–1. (a) Total (b) male (c) female.

The model performance for predicting ASD is presented in Table 2 and Table S2. The random forest model outperformed the logistic regression model at predicting ASD (accuracy: 0.996 vs. 0.686; AUC: 0.997 vs. 0.561; sensitivity: 0.554 vs. 0.989; specificity: 0.678 vs. 1.000). In the subgroup analysis, accuracy, AUC, and sensitivity were higher for females than males in both models. Figure 1 illustrates the clinical predictors based on random forest variable importance. For the total population, the major predictors of ASD were BMI (0.3679), SES (0.2164), maternal age at birth (0.1735), sex (0.0682), delivery institution (0.0549), cesarean delivery (0.0272), antidepressant use (0.017), gestational DM (0.0163), pregestational anxiety (0.0096), and PIH (0.009) (Figure 1a). Most predictors ranked among the top 10 for both subgroups (Figure 1b,c). Pregestational hypertension in males and FGR in females were among the new top 10 predictors.

**TABLE 2:** Ability of machine learning models to predict autism spectrum disorder.

Model	Accuracy			AUC			Sensitivity	Specificity
	Mean	CI‐L	CI‐U	Mean	CI‐L	CI‐U		
Total								
RF	0.996	0.995	0.997	0.997	0.996	0.998	0.989	1.000
Male								
RF	0.994	0.993	0.996	0.996	0.995	0.997	0.981	1.000
Female								
RF	0.999	0.998	0.999	0.999	0.998	1.000	0.996	1.000

Abbreviations: AUC, area under the receiver operating characteristic curve; CL‐L, lower bound of 95% confidence interval; CL‐U, upper bound of 95% confidence interval; RF, random forest.

**FIGURE 2:**
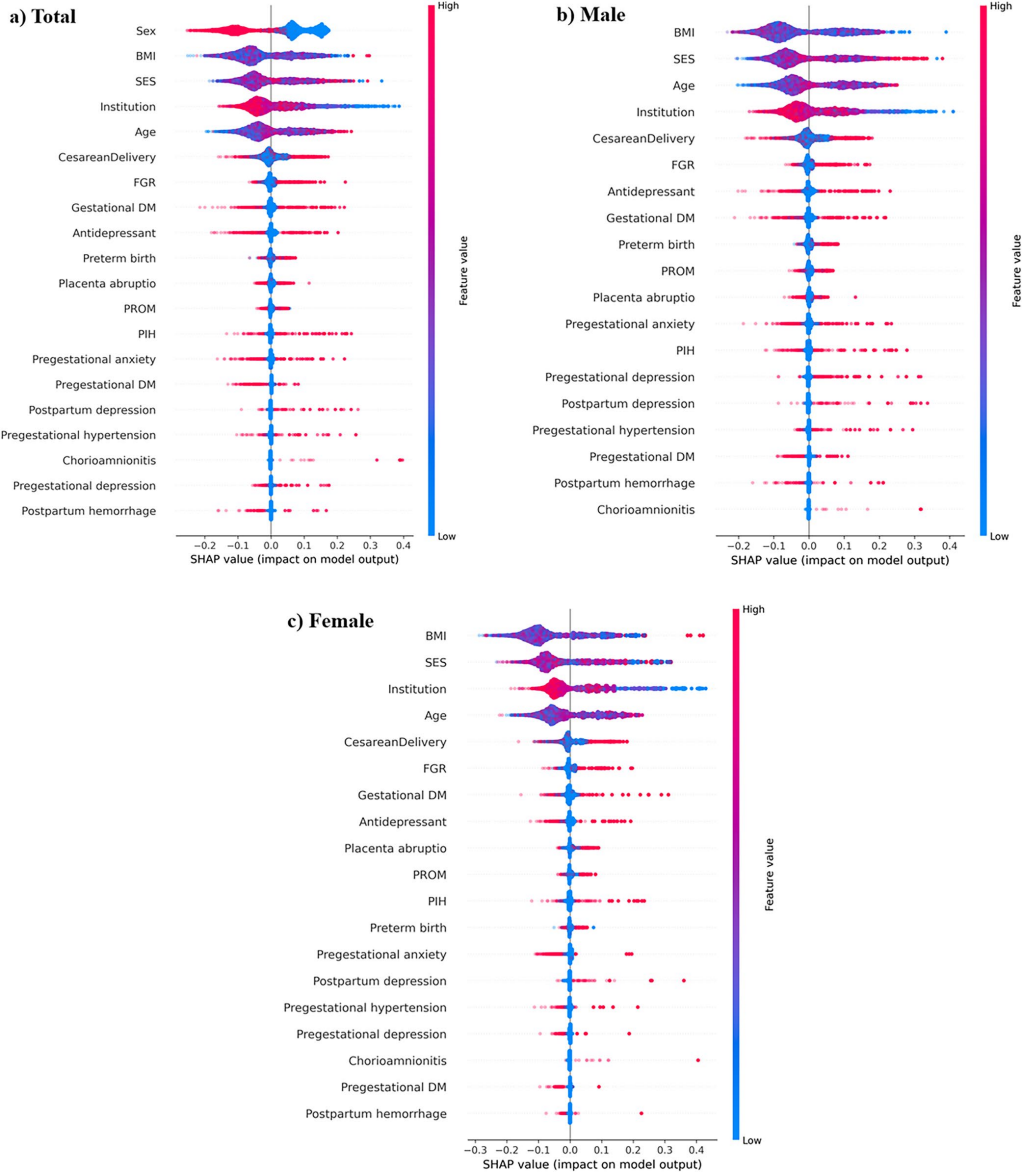
SHapley Additive exPlanations summary plot. (a) Total (b) male (c) female.

The positive association between ASD and its major predictors is illustrated by the SHAP values in Table S3. To assess this association, we compared the absolute values of the maximum and minimum SHAP values: a maximum that is greater than the minimum indicates a positive association; conversely, a maximum that is less than the minimum indicates a negative association. For example, SHAP values of SES for ASD range from −0.18183 to 0.329044. This indicates that SES can decrease or increase the probability of ASD. In this case, the absolute value of the maximum SHAP (0.329044) was greater than that of the minimum SHAP (−0.18183), indicating a positive association. Figure 2 shows the SHAP summary plot for ASD in the total population and by sex. In the total population, the features specific to ASD included sex (male) and institution (high level) (Figure 2a), both of which were negatively correlated with ASD prediction. Additionally, features such as FGR, antidepressant use, gestational DM, PIH, postpartum depression, pregestational hypertension, pregestational depression, postpartum hemorrhage, and chorioamnionitis were also associated with ASD prediction.

**FIGURE 3:**
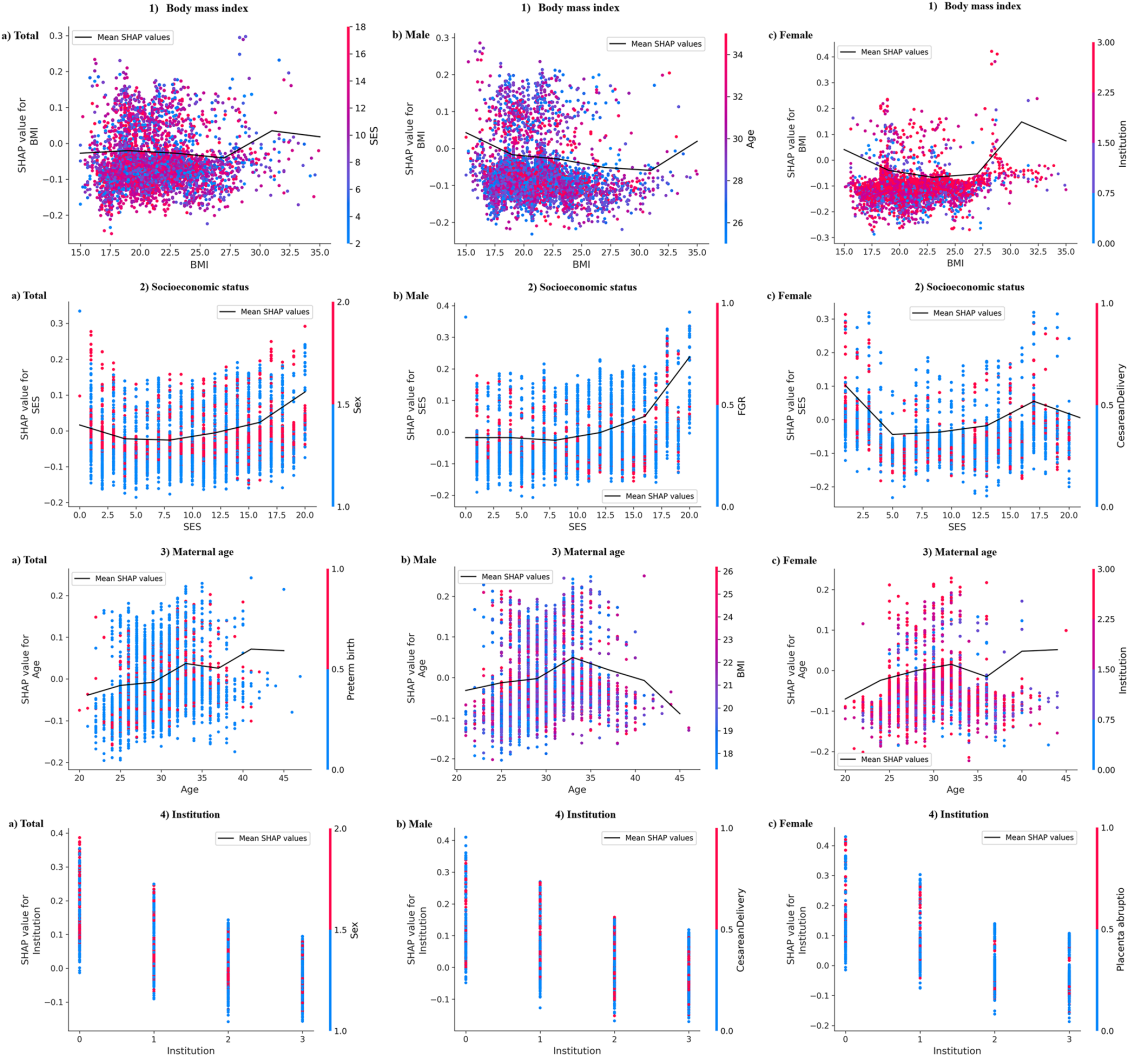
Shapley Additive Explanations dependence plot. (1) Body mass index: (a) total, (b) male, (c) female. (2) Socioeconomic status: (a) total, (b) male, (c) female. (3) Maternal age at birth: (a) total, (b) male, (c) female. (4) Institution level: (a) total, (b) male, (c) female.

Between the male and female groups, all features but BMI exhibited similar patterns (Figure 2b,c). In the male group, a lower BMI had a higher SHAP value, while in the female group, a higher BMI had a higher SHAP value. The impact of the top four factors on ASD risk prediction by the random forest model was further analyzed using SHAP dependence plots (Figure 3). A maternal BMI < 18.5 kg/m^2^ was linked to higher ASD risk in both sexes, while a maternal BMI ≥ 25.0 kg/m^2^ was associated with higher ASD risk in females (Figure 3‐1a–c). Additionally, a U‐shaped curve association was observed between SES and ASD, with an increased ASD risk for males with a lower SES and females with a higher SES (Figure 3‐2a–c).

Maternal age at birth was positively associated with the SHAP value for ASD (Figure 3‐3a–c). The SHAP value also increased with delivery institution level, showing higher values for more advanced hospitals (Figure 3‐4a–c). Figures S1 and S2 show the SHAP dependency plots for the other features and SHAP interaction plots. The SHAP interaction plot of two different predictors registers the SHAP summary plot of their interaction term, for example, BMI × SES. A wide spread of points in these plots indicates a strong interaction effect on ASD probability, while a narrow spread indicates a weak effect. For instance, the wide spread in the SHAP interaction plot for BMI and SES indicates a strong interaction effect on ASD probability. Conversely, BMI and chorioamnionitis showed a narrow spread, indicating a weaker interaction effect. Notably, the SHAP interaction plot for the same predictors on the vertical and horizontal axes reflects the SHAP summary plot of the predictor itself.

## 4 Discussion

In this study, we developed sex‐specific predictive models for ASD using prenatal and perinatal risk factors from KNHI data. The random forest models achieved high accuracy for the total population as well as the male and female groups. Major predictors included pregestational BMI, SES, maternal age at birth, sex, and delivery institution. These predictors were consistent across the male and female offspring groups. Notably, maternal pregestational BMI and SES had different effects on ASD risk between the male and female offspring.

A previous study reported a national‐level eight‐year cumulative prevalence of ASD in Korea of 5.2 per 1000 births in 2002 and 9.4 per 1000 births in 2012 (Yoo et al. 2022). Our data showed that the 14‐year cumulative prevalence of ASD among children born in 2007 was 10.3 per 1000 births. Although direct comparisons are challenging due to varying tracking periods, the prevalence of ASD in Korea seems to be increasing, a trend also observed in other studies. For example, the National Health Interview Survey in the USA reported that the prevalence of ASD among children aged 3–17 years increased from 1.1% to 2.5% between 2009 and 2017 (Zablotsky et al. 2019), rates that are higher than the prevalence in Korea. Systematic reviews from 2012 and 2022 reported a rise in global ASD prevalence from 0.62% in 2012 to 1.0% in 2021 (Zeidan et al. 2022; Elsabbagh et al. 2012). These trends likely reflect expanded diagnostic criteria, shifts in diagnoses from other developmental disorders to pervasive developmental disorders, improved access to services, greater awareness of ASD among the general public and professionals, and possibly increased risk factors associated with the disorder (Elsabbagh et al. 2012). As the medical and social burden of ASD grows, emphasis on promoting early and accurate diagnoses also increases.

This study found that the effect of maternal pregestational BMI on ASD varied between male and female offspring. Maternal underweight status was linked to increased risk of ASD for both sexes, while overweight or obesity status raised the risk only in female offspring. Maternal nutritional deficiencies could increase the risk of ASD, potentially influenced by insufficient intake of folic acid (Hoxha et al. 2021), multivitamins (DeVilbiss et al. 2017), and n‐3 polyunsaturated fatty acids, which are crucial for fetal brain development and powerful immunomodulators (Madore et al. 2016). Factors such as SES, healthy behaviors, or pregnancy characteristics that contribute to nutritional deficiencies might also play mediating roles.

Maternal obesity, identified as a significant risk factor for ASD, was reported in a previous meta‐analysis (Li et al. 2016). However, the finding that the impact of maternal obesity is more prominent in female offspring is inconsistent with previous research (Denizli et al. 2022). Several hypotheses might explain this discrepancy. First, maternal obesity, particularly during pregnancy, is associated with metabolic disruptions and an increased risk of obesity in the offspring, with evidence suggesting that females may be more susceptible to these effects (Heslehurst et al. 2019; Derraik et al. 2015), potentially affecting ASD risk. Second, excessive maternal weight gain may increase prenatal androgen exposure in female fetuses (Kallak et al. 2017), which could increase autistic‐like behaviors within them (Palomba et al. 2012; Gore et al. 2014). Third, maternal obesity is a recognized stressor on the developing fetal brain. One animal study demonstrated that maternal obesity reduces brain antioxidant defenses, impairs hippocampal *Bdnf* expression, and alters emotional behavior, with larger effects in female mice (Musillo et al. 2023). These sex‐specific effects of maternal obesity on brain development may also contribute to differences in ASD susceptibility. Future large‐scale human studies investigating the impact of maternal obesity on sex‐specific effects in the offspring are warranted.

This study found a U‐shaped relationship between SES and ASD risk, with increased risk in males with a lower SES and females with a higher SES. Studies conducted in the United States consistently reported that the prevalence of ASD increased with increasing parental SES (Durkin et al. 2010, 2017; Thomas et al. 2012). A recent study in Taiwan reported similar findings (Yu et al. 2021). Conversely, studies in Denmark and the United Kingdom found no association between SES and ASD (Larsson et al. 2005; Kelly et al. 2019; Sun et al. 2014), while studies in Sweden and France reported that a lower parental SES was associated with a higher risk of ASD (Rai et al. 2012; Delobel‐Ayoub et al. 2015). These results may be due to variations in public healthcare systems, medical infrastructure, and ASD screening practices across systems. The association between a higher SES and ASD may be explained by the higher SES being linked to older maternal age at marriage and childbirth (Karney 2021), greater awareness of ASD, and better access to healthcare services, factors that facilitate an early diagnosis (Fountain et al. 2011). However, a low SES might be linked to a higher ASD risk due to increased genetic vulnerability, exposure to environmental toxins, and social stressors (Rai et al. 2012). Further research is needed to investigate the sex‐specific mechanisms that underlie the varying impacts of SES on ASD risk.

The impact of advanced maternal age at birth on the risk of ASD has been debated (King et al. 2009; Lung et al. 2018). This study confirmed that advanced maternal age was associated with a higher risk of ASD. The influence of maternal age on the risk of ASD may vary by offspring sex (Croen et al. 2007; Sandin et al. 2012). For example, one historical birth cohort study found that advanced maternal age had a stronger impact on ASD in female offspring (Croen et al. 2007), while a meta‐analysis reported a clearer association in studies with a higher proportion of male offspring (Sandin et al. 2012). In this study, maternal age was a significant risk factor for ASD in both sexes. Specifically, for males, the risk of ASD increased with maternal age < 35 years, whereas for females, the risk increased with maternal age > 35 years. This finding contrasts with that of Lung et al. who indicated that the risk of a child being diagnosed with ASD increased if the mother was > 40 years old (Lung et al. 2018). Several mechanisms may explain how maternal age influences the risk of ASD. First, advanced maternal age may contribute to the genetic mutations associated with ASD.

Maternal age at conception could influence the *de novo* mutation rate because of the accumulation of damage in oocytes and potentially via influence on the number of postzygotic mutations in the embryo (Gao et al. 2019). These germline mutations appear to accumulate roughly to the absolute time of maternal age. Second, maternal aging during pregnancy could lead to enduring changes in the epigenetic characteristics of the DNA methylation of the child that impact their health, including the development of neurodevelopmental disorders such as ASD (Markunas et al. 2016). Third, as maternal age increases, exposure accumulates to environmental toxins such as lead, polychlorinated biphenyls, marijuana, alcohol, and tobacco, which can adversely affect the long‐term neurodevelopment of the offspring (Williams and Ross 2007). Additionally, prenatal exposure to particulate matter reportedly influences ASD through neuropathological mechanisms such as neuroinflammation, mitochondrial disruptions, oxidative stress, and epigenetic changes (Liu et al. 2023). Advanced maternal age may increase exposure to particulate matter, potentially affecting ASD occurrence and severity.

Delivery institution was an important predictive factor for ASD. Higher hospital levels correlated with an increased risk of ASD in both sexes. The highest hospital level is the tertiary general hospital, which provides advanced medical care for serious conditions. Therefore, a delivery at a tertiary general hospital suggests that the mother likely required more complex treatment and care due to prenatal or pregnancy‐related comorbidities (Kim and Choi 2016). In 2014, South Korea established Integrated Care Centers for High‐Risk Mothers and Newborns in each region (Ahn et al. 2018), primarily at tertiary hospitals. These centers facilitate the transfer of high‐risk mothers and newborns to receive intensive care. The increased risk of ASD associated with a higher‐level hospital ultimately reflects that newborns of high‐risk pregnancies are more likely to develop ASD, correlating with maternal risk factors such as GDM, PIH, and postpartum hemorrhage, and neonatal risk factors such as preterm birth and FGR.

This study has some limitations. First, the diagnoses of ASD were based on ICD‐10 codes extracted from the KNHIS database rather than being confirmed through specific diagnostic tools. This approach may affect diagnostic accuracy and did not fully account for variations in ASD severity. While validation studies focused specifically on ASD diagnoses within this database are limited, recent research on the accuracy of diagnoses derived from insurance claims data has reported a sensitivity range of 78.1%–88.7% and a specificity of 100% when compared with medical records (Lee et al. 2020). Nevertheless, we recognize the necessity for further validation studies specifically addressing ASD diagnoses within the NHIS database to enhance the reliability of such data. Second, despite potential differences in risk factors and pathophysiology depending on whether ASD is accompanied by intellectual disability, identifying the presence of intellectual disability in this database was challenging. Third, this study included only 14 years of data follow‐up after birth and excluded cases of adult ASD diagnosed after adolescence. Fourth, despite paternal and maternal age being important risk factors for ASD (Lyall et al. 2020), this database did not include information on paternal age. Fifth, the data used in this study is based on the KNHI claim database, which is limited to a specific population. Therefore, the generalizability and performance of the model may be restricted. Future research should consider using data from other countries or diverse populations to externally validate the model, thereby extending the findings and evaluating its performance in various contexts. Sixth, the sample size for females in the training set is less than 150. A smaller sample size can increase the risk of overfitting and reduce the robustness of the model. However, it is worth noting that in our analysis, the accuracy, AUC, and sensitivity were notably higher for females compared to males. Despite the smaller sample size for females, the model showed strong predictive performance. To address this limitation, we conducted extensive validation within the available dataset and ensured that the model performed consistently across both male and female groups. Nonetheless, we recognize the need for larger sample sizes to further assess the model's generalizability and stability. Future studies with larger and more balanced datasets would provide a more comprehensive evaluation of the model's performance. Seventh, it can be noted that pregestational BMI was designed as a continuous variable in this study, but different designs of this variable as categorical variables can bring different results.

## 5 Conclusion

This study identified pregestational BMI, SES, maternal age at birth, sex, and delivery institution as significant predictors of ASD. Maternal pregestational BMI and SES exhibited varying effects on ASD between male and female offspring. Understanding these sex‐specific predictors is crucial for enabling prevention, early diagnosis, and intervention strategies tailored to each sex. Further research and comprehensive data analyses are necessary to emphasize the importance of individualized approaches to ASD management.

## Author Contributions

Full access to all data in the study, responsibility for the integrity of the data, and the accuracy of the data analysis: Kwang‐Sig Lee, Ki Hoon Ahn. Concept and design: Ju Sun Heo, Seung‐Woo Yang, Ki Hoon Ahn. Acquisition, analysis, or interpretation of the data: All authors. Drafting of the manuscript: Ju Sun Heo. Critical revision of the manuscript for important intellectual content: Ju Sun Heo, Seung‐Woo Yang, Kwang‐Sig Lee, Ki Hoon Ahn. Statistical analysis: Sohee Lee, Kwang‐Sig Lee. Funding: Ju Sun Heo, Seung‐Woo Yang, Kwang‐Sig Lee, Ki Hoon Ahn. Administrative, technical, or material support: Sohee Lee, Kwang‐Sig Lee. Supervision: Ju Sun Heo, Seung‐Woo Yang, Kwang‐Sig Lee, and Ki Hoon Ahn.

## Conflicts of Interest

The authors declare no conflicts of interest.

## Supporting information


Data S1.

## Data Availability

The data that support the findings of this study are available on request from the corresponding author. The data are not publicly available due to privacy or ethical restrictions.
