# Supplementary material for: Sex‐Based Differences in Prenatal and Perinatal Predictors of Autism Spectrum Disorder Using Machine Learning With National Health Data
Source: Autism Res. 2025 May 19;18(7):1330–41. doi: 10.1002/aur.70054 (PMC12279001; doi:10.1002/aur.70054)
Supplement: Supplementary file 1 — Data S1. [file AUR-18-1330-s001.docx]

**Autism Research**

**Sex-Based Differences in Prenatal and Perinatal Predictors of** **Autism Spectrum Disorder using Machine Learning with National Health Data**

Ju Sun Heo, MD, PhD^1,2*^, Seung-Woo Yang, MD, PhD^3,4*^, Sohee Lee^5,6,7^, Kwang-Sig Lee, PhD^5**^, Ki Hoon Ahn, MD, PhD^7**^

^1^Department of Pediatrics, Seoul National University College of Medicine, Seoul, Korea

^2^Department of Pediatrics, Seoul National University Children’s Hospital, Seoul, Korea

^3^Research Institute of Medical Science, Konkuk University School of Medicine, Seoul, Korea

^4^Sanford Consortium for Regenerative Medicine, School of Medicine, University of California, San Diego, CA 92037, USA

^5^AI Center, Korea University College of Medicine, Anam Hospital, Seoul, Korea

^6^Department of Statistics, Korea University College of Political Science and Economics, Seoul, Korea

^7^Department of Obstetrics and Gynecology, Korea University College of Medicine, Anam Hospital, Seoul, Korea

*Ju Sun Heo and Seung-Woo Yang contributed equally to this work.

**Corresponding Authors:

Kwang-Sig Lee, PhD

AI Center, Korea University College of Medicine, Anam Hospital

73 Goryeodae-ro, Seongbuk-gu, Seoul 02841, Korea

E-mail: ecophy@hanmail.net

Ki Hoon Ahn, MD, PhD

Department of Obstetrics & Gynecology, Korea University College of Medicine, Anam Hospital

73 Goryeodae-ro, Seongbuk-gu, Seoul 02841, Korea

E-mail: akh1220@hanmail.net

**Supplementary Table 1. ICD-10 codes for study variables**

| Variable | Code | Description |
| --- | --- | --- |
| Autism spectrum disorder | F84.0 | Childhood autism |
|  | F84.1 | Atypical autism |
|  | F84.4 | Overactive disorder associated with mental retardation and stereotyped movements |
|  | F84.5 | Asperger syndrome |
|  | F84.8 | Other pervasive developmental disorders |
|  | F84.9 | Pervasive developmental disorder, unspecified |
| Preterm birth | O42.0 | Premature rupture of membranes, onset of labor within 24 h |
|  | O42.1 | Premature rupture of membranes, onset of labor after 24 h |
|  | O42.2 | Premature rupture of membranes, labor delayed by therapy |
|  | O42.9 | Premature rupture of membranes, unspecified |
|  | O60.1 | Preterm spontaneous labor with preterm delivery |
|  | O60.3 | Preterm delivery without spontaneous labor |
| Pregestational hypertension | I10-I15 | Hypertensive diseases |
| Pregestational DM | E10-E14 | Diabetes mellitus |
|  | O24.0 | Pre-existing type 1 diabetes mellitus |
|  | O24.1 | Pre-existing type 2 diabetes mellitus |
|  | O24.2 | Pre-existing malnutrition-related diabetes mellitus |
|  | O24.3 | Pre-existing diabetes mellitus, unspecified |
| Pregestational depression | F32 | Depressive episode |
|  | F33 | Recurrent depressive disorder |
|  | F34 | Persistent mood [affective] disorders |
|  | F38 | Other mood [affective] disorders |
|  | F39 | Unspecified mood [affective] disorder |
| Pregestational anxiety | F40 | Phobic anxiety disorders |
|  | F41 | Other anxiety disorders |
|  | F44 | Dissociative [conversion] disorders |
|  | F45 | Somatoform disorders |
|  | F48 | Other neurotic disorders |
| Gestational DM | O24.4 | Diabetes mellitus arising in pregnancy |
|  | O24.9 | Diabetes mellitus in pregnancy, unspecified |
| PIH | O13 | Gestational [pregnancy-induced] hypertension |
|  | O14 | Pre-eclampsia |
|  | O15 | Eclampsia |
|  | O16 | Unspecified maternal hypertension |
| Chorioamnionitis | O41.1 | Infection of amniotic sac and membranes |
| Placental abruption | O45 | Premature separation of placenta [abruptio placentae] |
| PROM | O42.0 | Premature rupture of membranes, onset of labor within 24 h |
|  | O42.1 | Premature rupture of membranes, onset of labor after 24 h |
|  | O42.2 | Premature rupture of membranes, labor delayed by therapy |
|  | O42.9 | Premature rupture of membranes, unspecified |
| Fetal growth restriction | O36.5 | Maternal care for poor fetal growth |
| Antidepressant medication | N06A  (ATC code) | Antidepressants |
| Postpartum hemorrhage | O72.0 | Third-stage hemorrhage |
|  | O72.1 | Other immediate postpartum hemorrhage |
|  | O72.2 | Delayed and secondary postpartum hemorrhage |
|  | O72.3 | Postpartum coagulation defects |
| Postpartum depression | F32 | Depressive episode |
|  | F33 | Recurrent depressive disorder |
|  | F34 | Persistent mood [affective] disorders |
|  | F38 | Other mood [affective] disorders |
|  | F39 | Unspecified mood [affective] disorder |
|  | F53 | Mental and behavioral disorders associated with the puerperium, NEC |

ATC, Anatomical Therapeutic Chemical classification system; DM, diabetes mellitus; ICD-10, International Statistical Classification of Diseases, 10^th^ revision; NEC, not elsewhere classified; PIH, pregnancy-induced hypertension; PROM, pre-labor rupture of membranes

**Supplementary Table 2. Logistic regression models to predict autism spectrum disorder**

|  | **Accuracy** | | |  | **AUC** | | | **Sensitivity** | **Specificity** |
| --- | --- | --- | --- | --- | --- | --- | --- | --- | --- |
| **Model** | **Mean** | **CI-L** | **CI-U** |  | **Mean** | **CI-L** | **CI-U** |  |  |
| **Total** |  |  |  |  |  |  |  |  |  |
| LR | 0.686 | 0.680 | 0.692 |  | 0.568 | 0.561 | 0.574 | 0.554 | 0.678 |
| **Male** |  |  |  |  |  |  |  |  |  |
| LR | 0.676 | 0.666 | 0.683 |  | 0.519 | 0.509 | 0.528 | 0.585 | 0.684 |
| **Female** |  |  |  |  |  |  |  |  |  |
| LR | 0.707 | 0.699 | 0.716 |  | 0.588 | 0.580 | 0.598 | 0.636 | 0.711 |

AUC, area under the receiver operating characteristic curve; CL-L, lower bound of 95% confidence interval; CL-U, upper bound of 95% confidence interval; LR, logistic regression

**Supplementary Table 3. Random forest Shapley Addictive Explanations values**

|  | **Total** | | |  | **Male** | | |  | **Female** | | |
| --- | --- | --- | --- | --- | --- | --- | --- | --- | --- | --- | --- |
| **Variable** | **Mean** | **Min** | **Max** |  | **Mean** | **Min** | **Max** |  | **Mean** | **Min** | **Max** |
| SES | -0.00528 | -0.18183 | 0.329044 |  | -0.00162 | -0.19692 | 0.357214 |  | -0.00342 | -0.25954 | 0.347881 |
| Age | 0.00127 | -0.18278 | 0.273619 |  | 0.007373 | -0.21186 | 0.295627 |  | -0.01021 | -0.2485 | 0.221546 |
| BMI | -0.02153 | -0.22053 | 0.326356 |  | -0.02806 | -0.26651 | 0.361115 |  | -0.04279 | -0.33434 | 0.466997 |
| Sex | 0.017279 | -0.24146 | 0.175748 |  |  |  |  |  |  |  |  |
| Institution | 0.014151 | -0.16104 | 0.366995 |  | 0.01951 | -0.1655 | 0.407894 |  | 0.033015 | -0.18068 | 0.424855 |
| FGR | 0.004649 | -0.06112 | 0.177147 |  | 0.006257 | -0.05781 | 0.237818 |  | 0.008968 | -0.09319 | 0.215886 |
| PROM | 0.001312 | -0.03984 | 0.059609 |  | 0.002892 | -0.05151 | 0.087275 |  | 0.003698 | -0.03983 | 0.076287 |
| Placenta abruptio | 0.001803 | -0.07311 | 0.065946 |  | 0.003285 | -0.05462 | 0.098168 |  | 0.004632 | -0.05095 | 0.070844 |
| Postpartum depression | 0.000505 | -0.03311 | 0.31824 |  | 0.000161 | -0.06556 | 0.34801 |  | 0.000031 | -0.05051 | 0.226187 |
| Antidepressant | 0.00175 | -0.17031 | 0.206465 |  | 0.002699 | -0.19823 | 0.245407 |  | 0.002156 | -0.17377 | 0.20983 |
| Gestational DM | 0.002212 | -0.1693 | 0.265189 |  | 0.001467 | -0.23344 | 0.220214 |  | 0.002993 | -0.12083 | 0.2768 |
| PIH | 0.000353 | -0.1001 | 0.22471 |  | 0.000126 | -0.15077 | 0.227606 |  | 0.000869 | -0.12058 | 0.230284 |
| Pregestational anxiety | 0.000742 | -0.18615 | 0.164366 |  | 0.00157 | -0.13092 | 0.267213 |  | 0.000996 | -0.12631 | 0.186295 |
| Pregestational depression | 0.00032 | -0.114 | 0.211358 |  | 0.00085 | -0.08854 | 0.310217 |  | -7.409810 | -0.07233 | 0.205959 |
| Pregestational DM | 0.000155 | -0.10907 | 0.101285 |  | 0.000435 | -0.10418 | 0.084812 |  | 0.000336 | -0.13937 | 0.083079 |
| Pregestational hypertention | 0.000046 | -0.1144 | 0.231273 |  | 0.000322 | -0.12443 | 0.215262 |  | 0.000251 | -0.08916 | 0.179894 |
| Preterm birth | 0.001614 | -0.07182 | 0.081171 |  | 0.000209 | -0.03947 | 0.066025 |  | 0.002678 | -0.06927 | 0.083646 |
| Chorioamnionitis | -0.000046 | -0.01712 | 0.329516 |  | 0.000040 | -0.04092 | 0.28784 |  | -0.000012 | -0.0155 | 0.320853 |
| Postpartum hemorrhage | 0.000159 | -0.20743 | 0.215803 |  | 0.000077 | -0.22421 | 0.196983 |  | 0.000276 | -0.04946 | 0.224386 |
| Cesarean delivery | 0.006218 | -0.17273 | 0.14699 |  | 0.009039 | -0.16473 | 0.175778 |  | 0.007894 | -0.20516 | 0.174903 |

Abbreviations

BMI, body mass index; DM, diabetes mellitus; FGR, fetal growth restriction; PIH, pregnancy-induced hypertension; PROM, pre-labor rupture of membrane; SES, socioeconomic status

**Supplementary Figure 1. Shapley Addictive Explanations dependence plot**

1. **Cesarean delivery**

**a) Total b) Male c) Female**


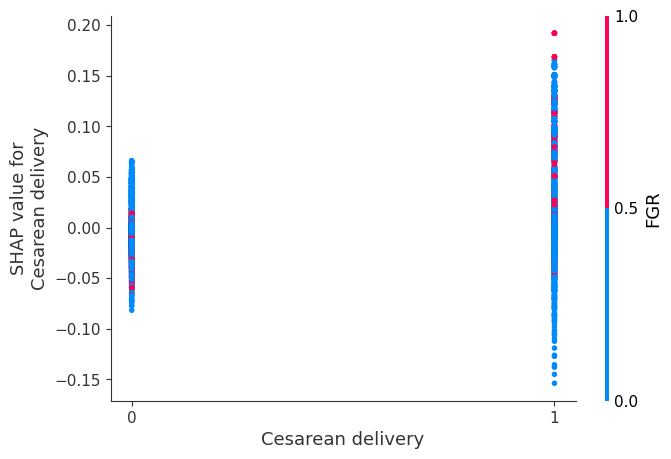

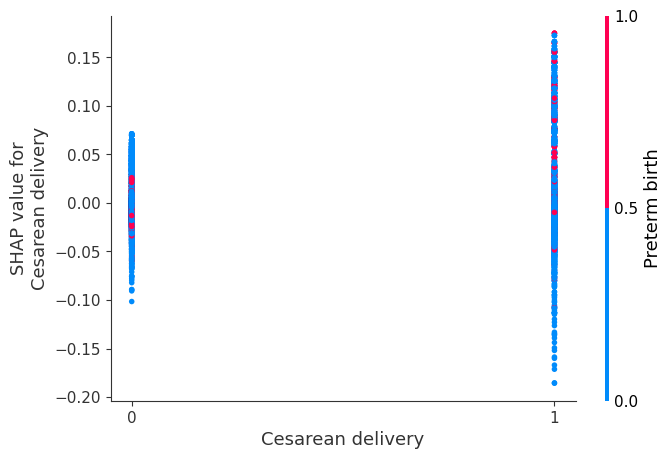

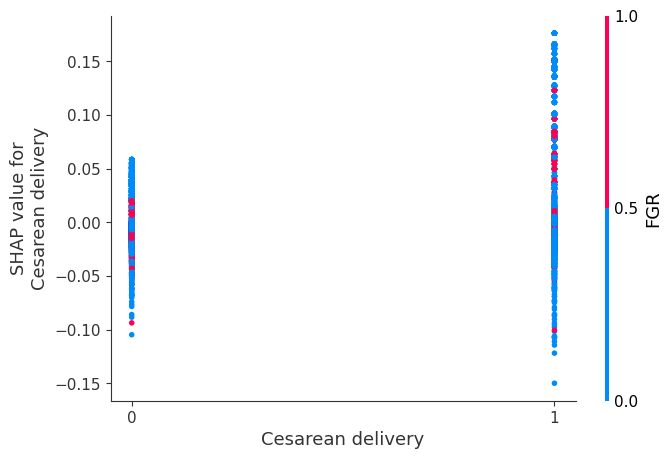


1. **Antidepressant**

**a) Total b) Male c) Female**


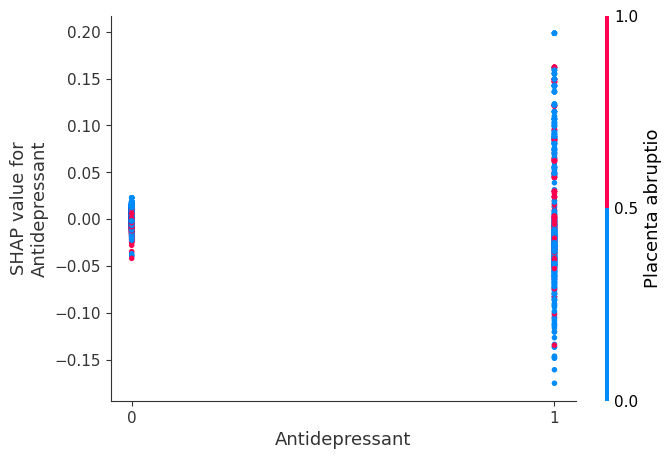

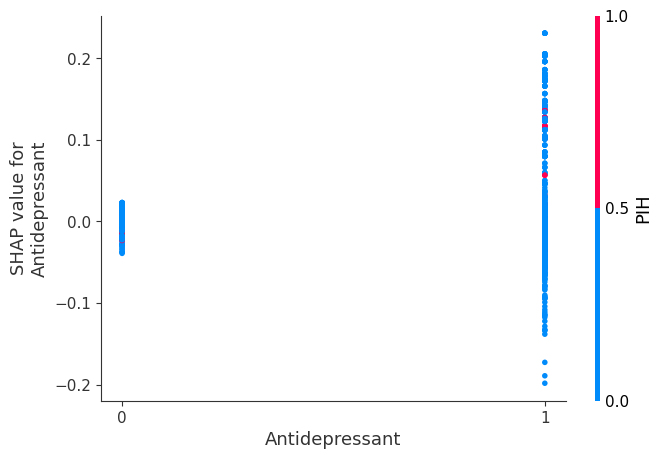

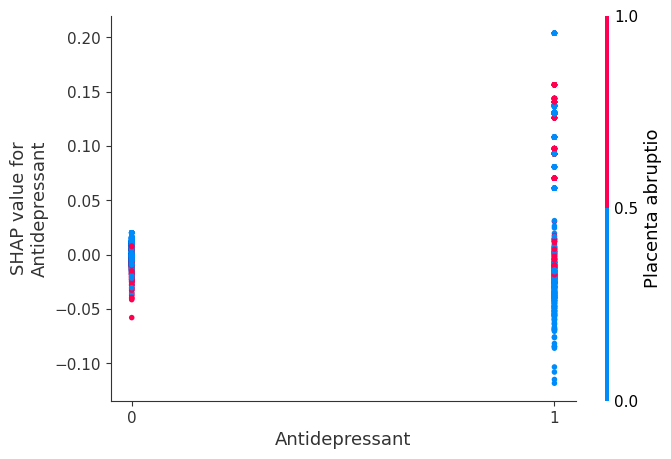


1. **Gestational diabetes mellitus**

**a) Total b) Male c) Female**


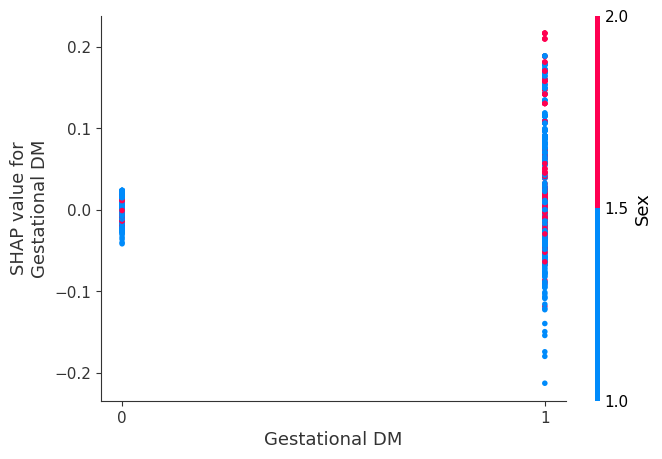

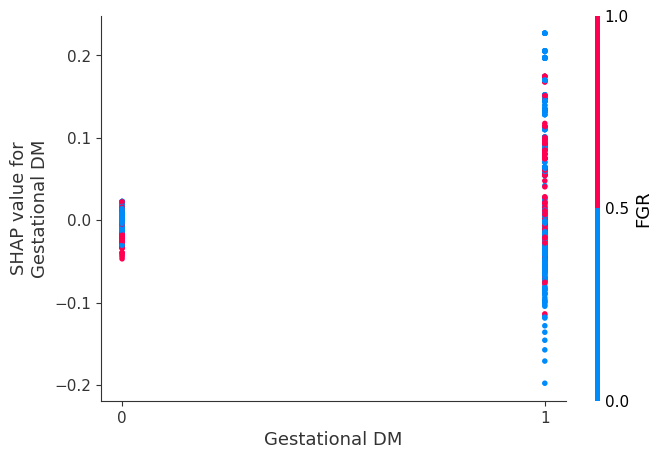

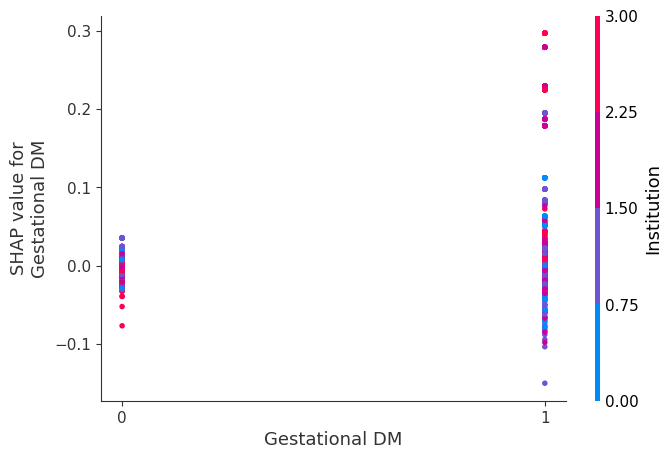


1. **Pregestational anxiety**

**a) Total b) Male c) Female**


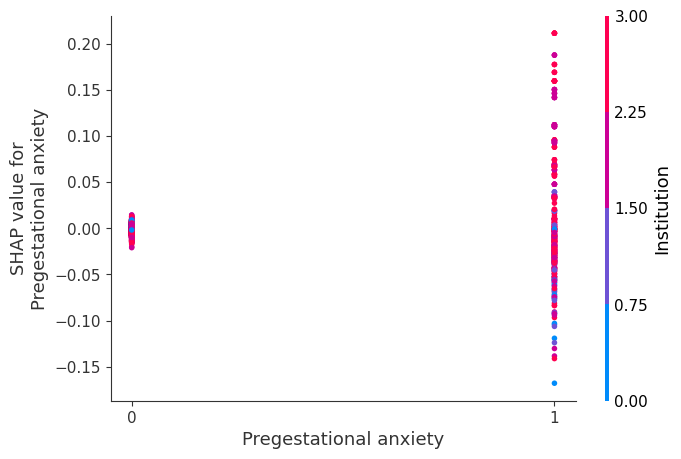

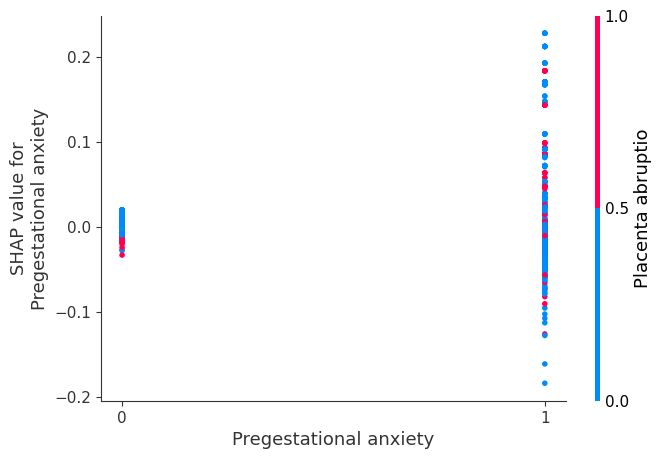

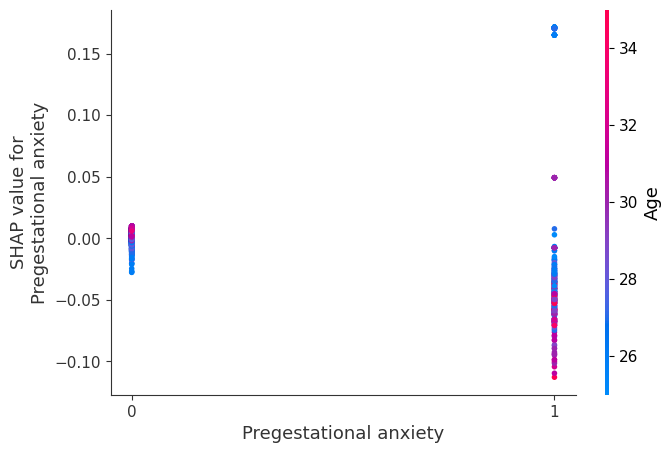


1. **Pregnancy-induced hypertension**

**a) Total b) Male c) Female**


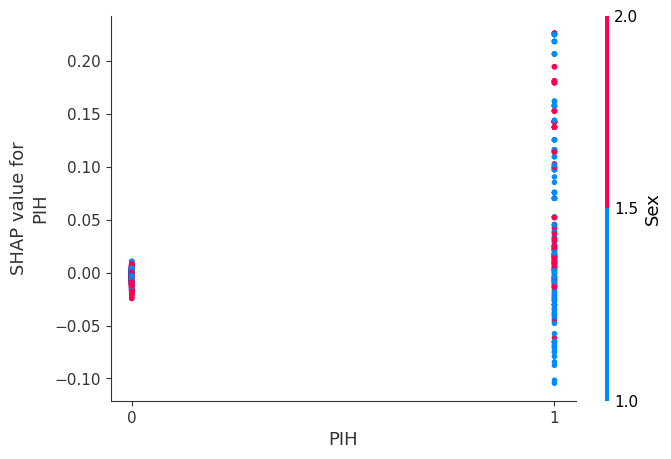

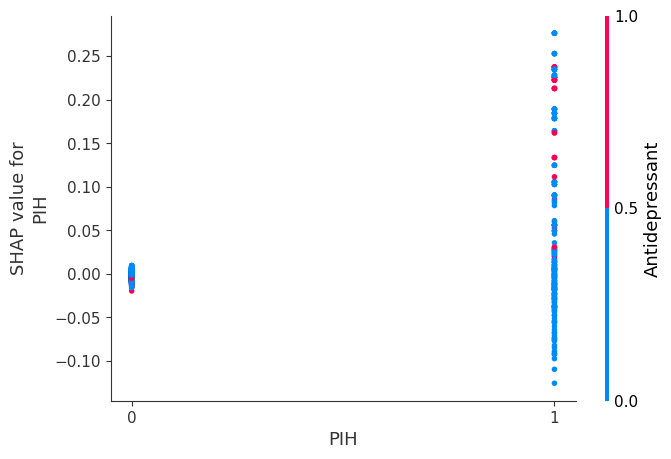

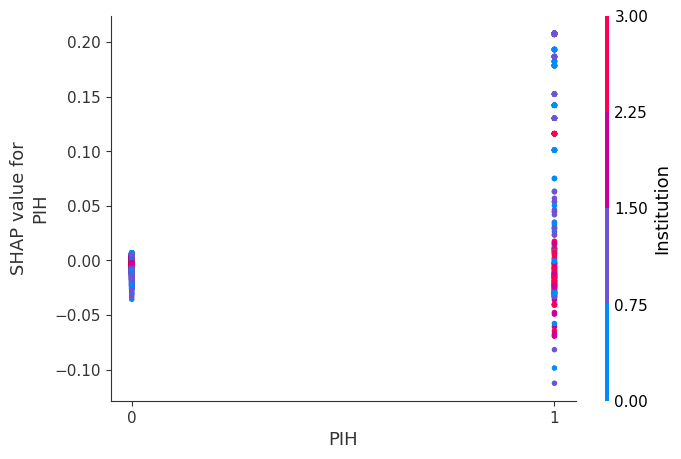


1. **Pregestational hypertension**

**a) Total b) Male c) Female**


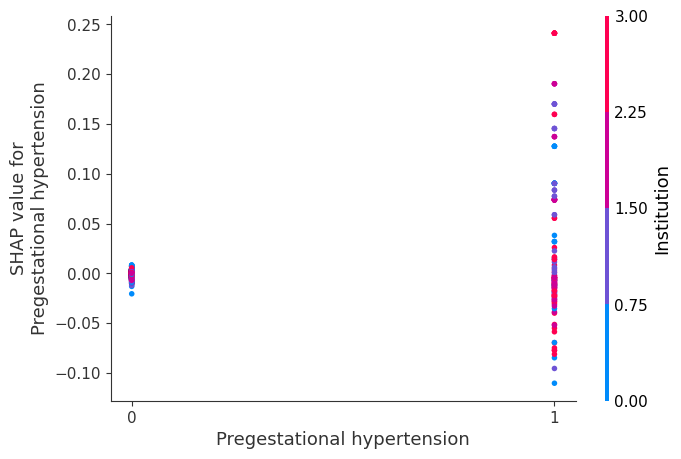

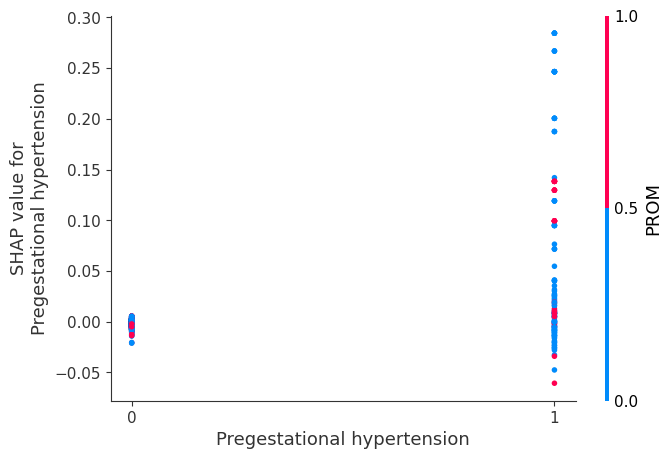

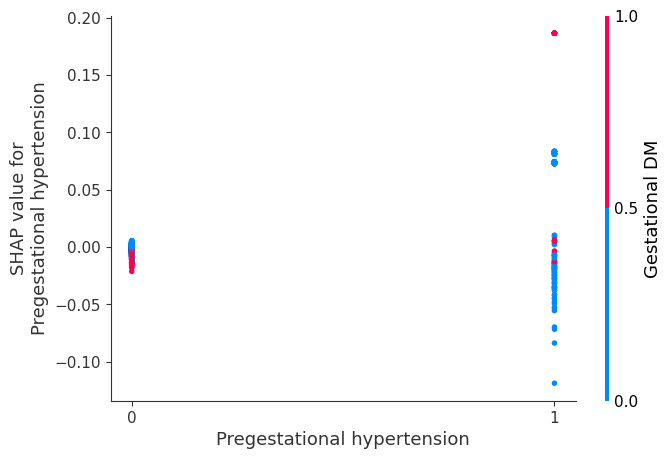


1. **Fetal growth restriction**

**a) Total b) Male c) Female**


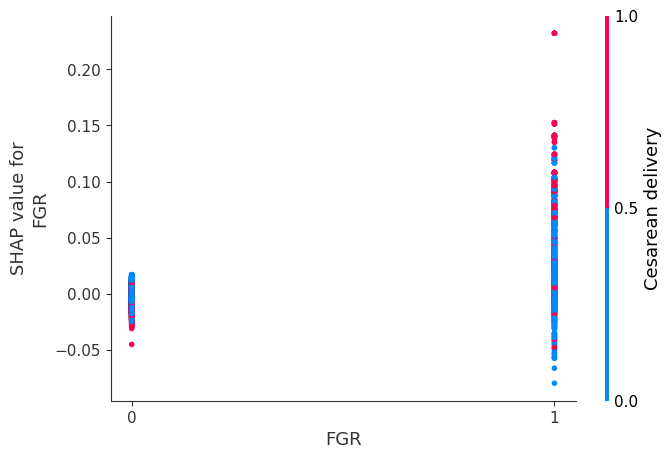

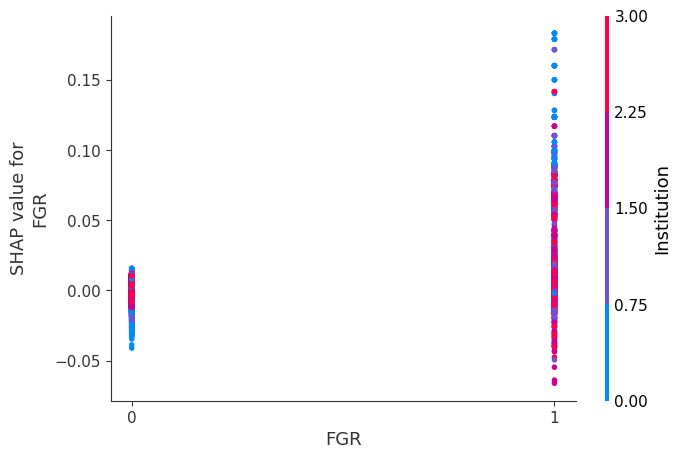

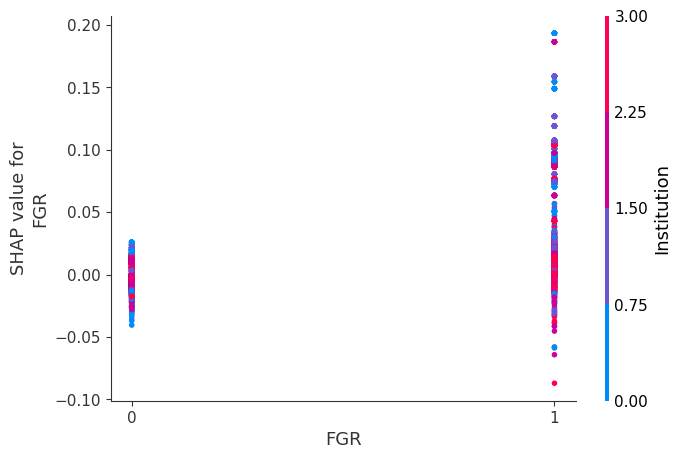


1. **Pregestational depression**

**a) Total b) Male c) Female**


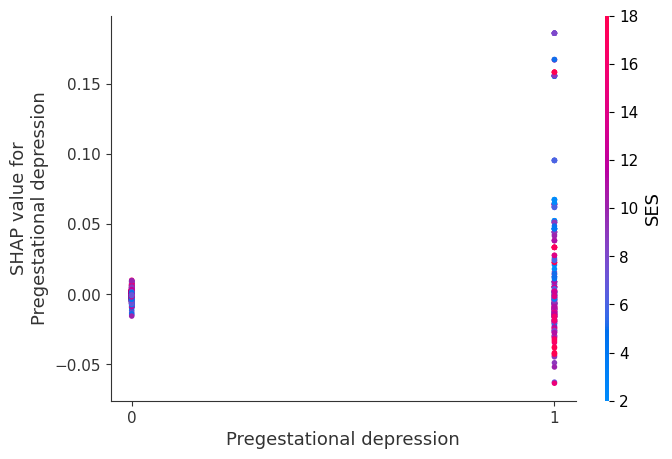

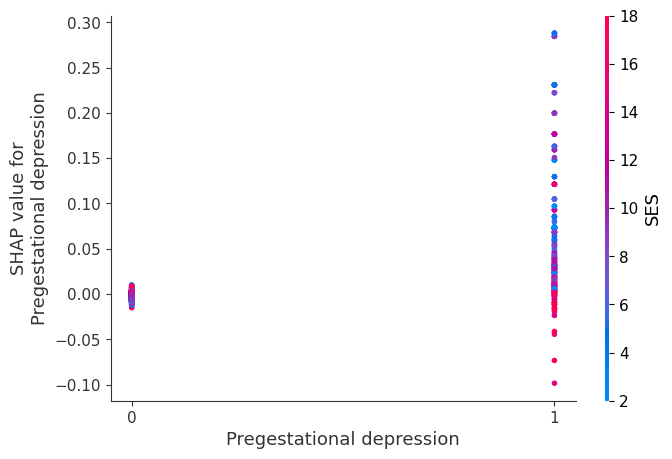

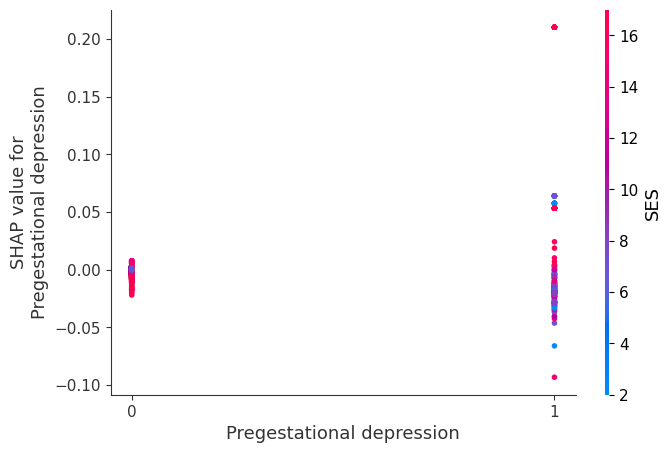


1. **Postpartum depression**

**a) Total b) Male c) Female**


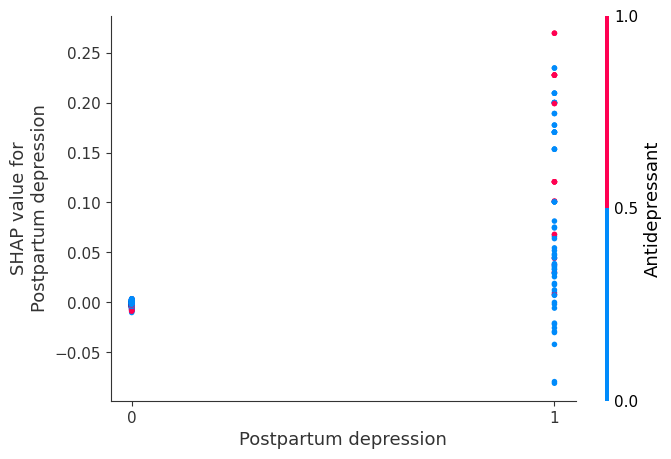

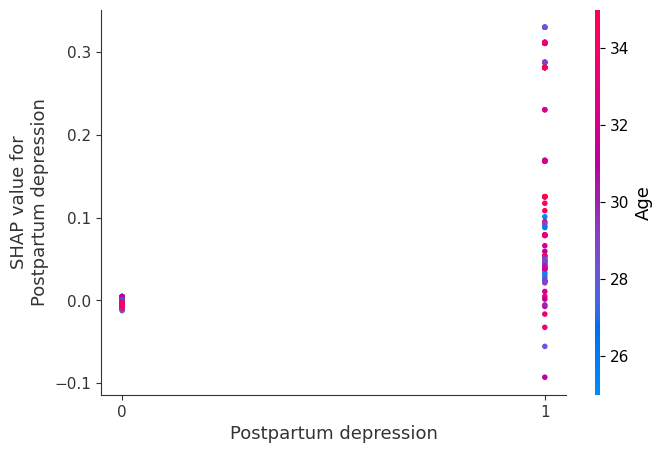

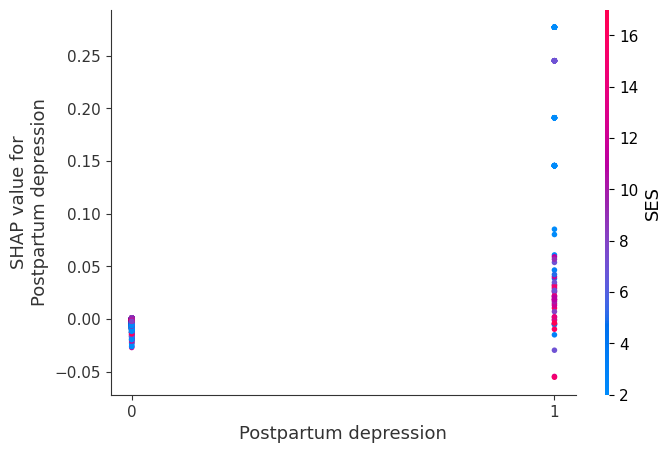


1. **Pregestational diabetes mellitus**

**a) Total b) Male c) Female**


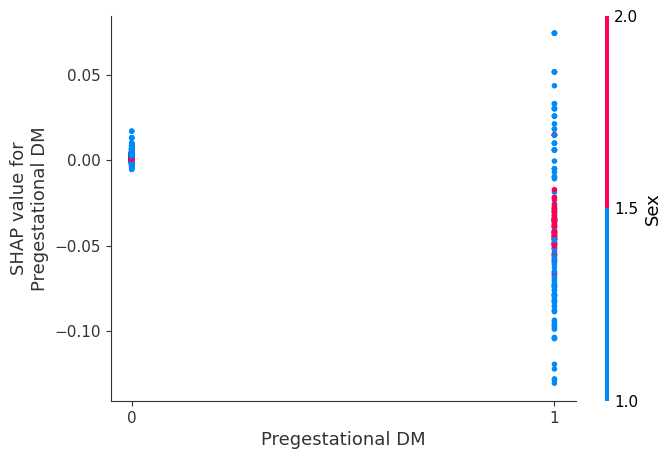

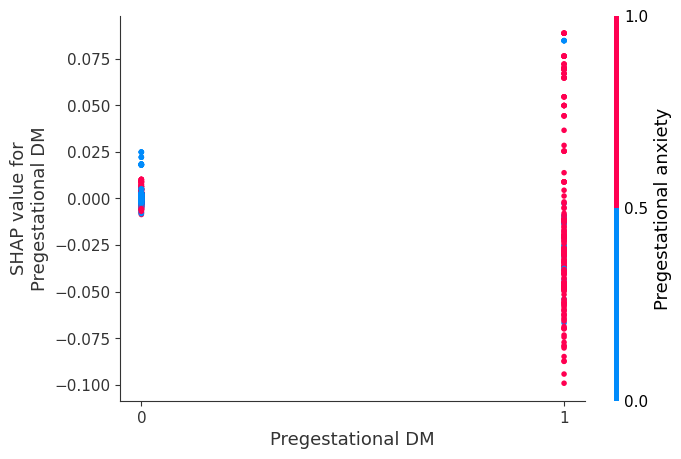

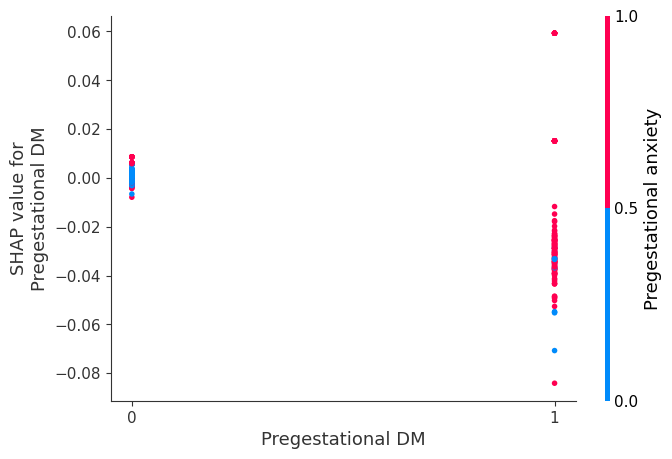


1. **Postpartum hemorrhage**

**a) Total b) Male c) Female**


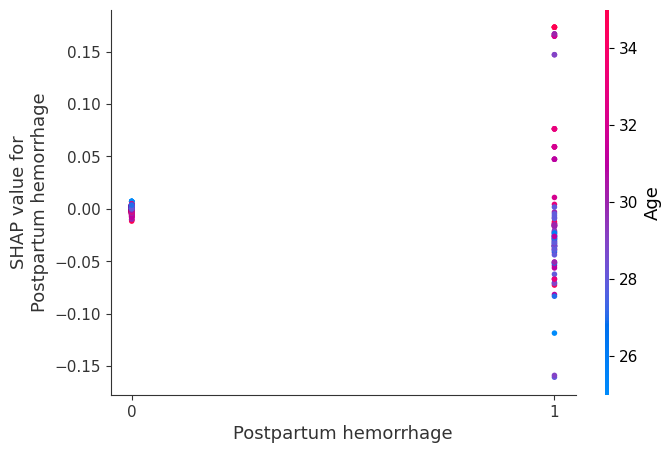

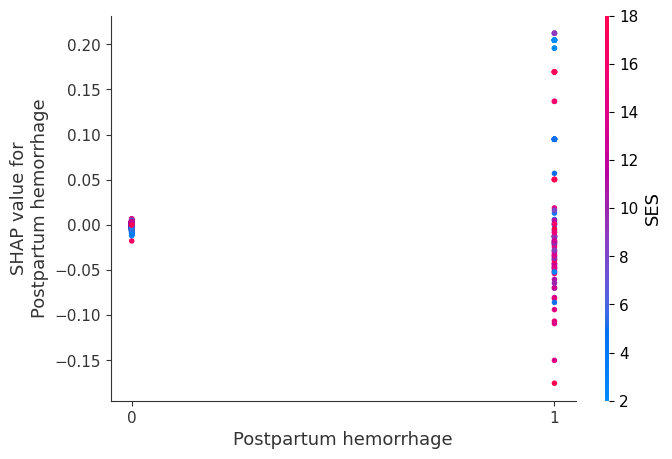

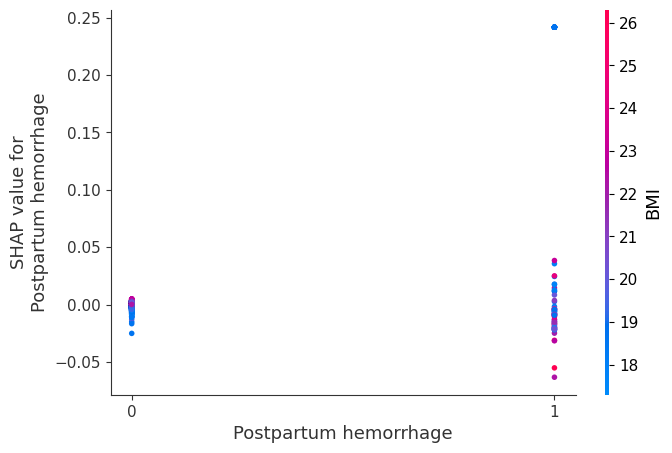


1. **Preterm birth**

**a) Total b) Male c) Female**


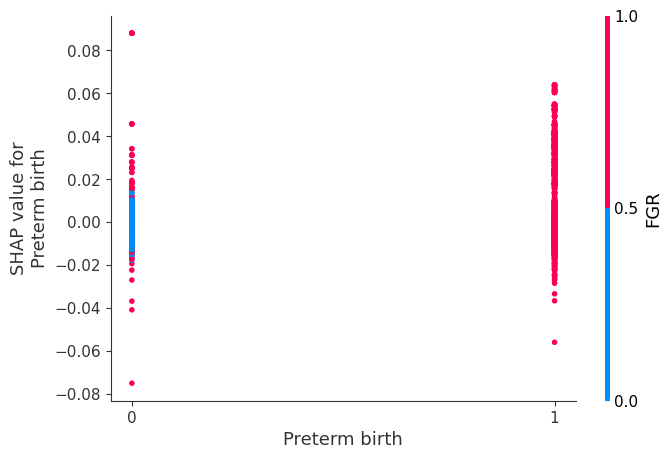

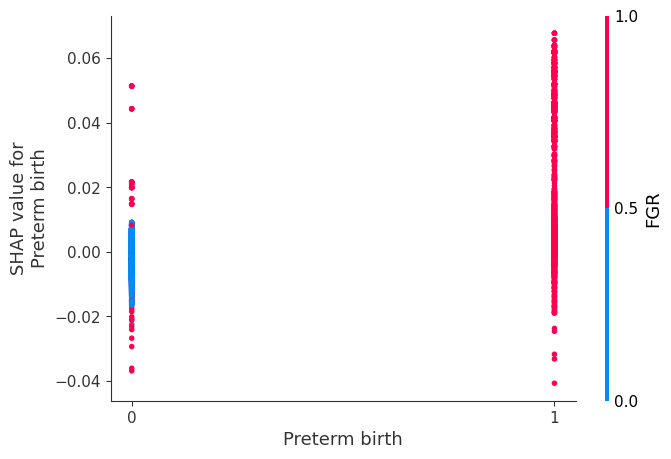

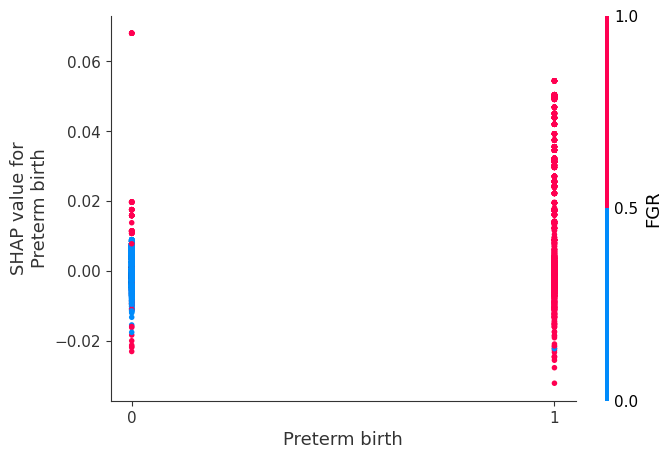


1. **Placenta abruptio**

**a) Total b) Male c) Female**


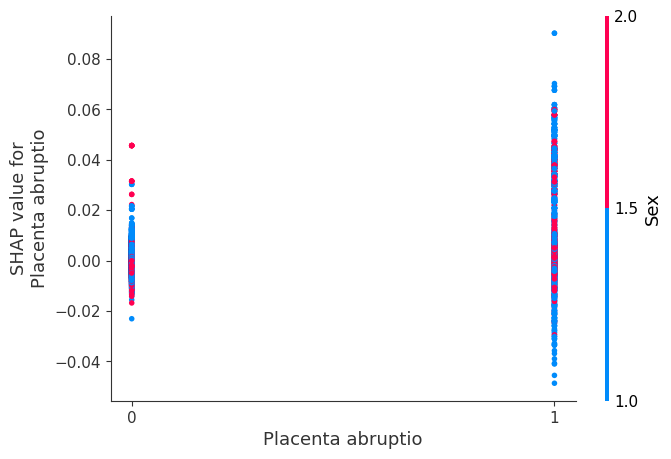

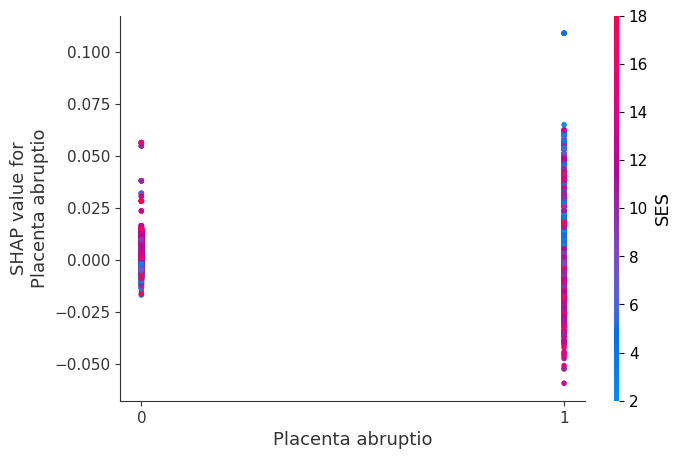

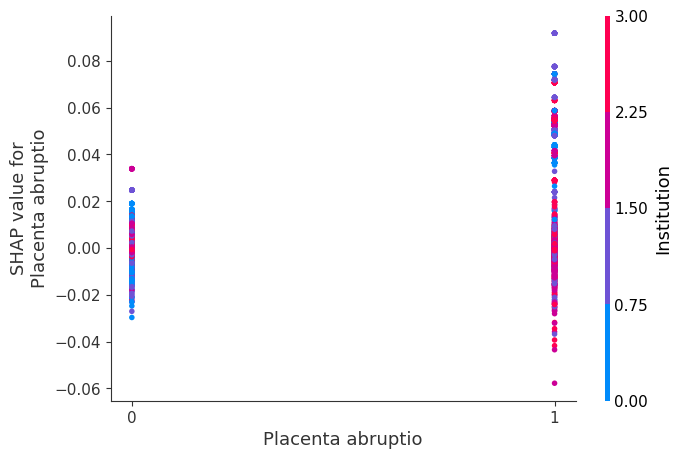


1. **Pre-labor rupture of membrane**

**a) Total b) Male c) Female**


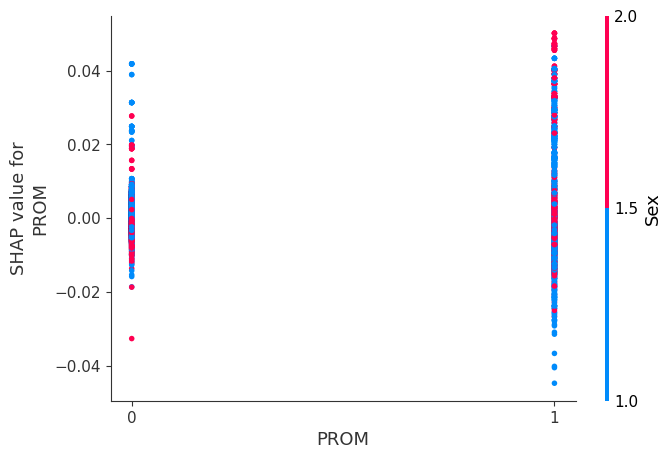

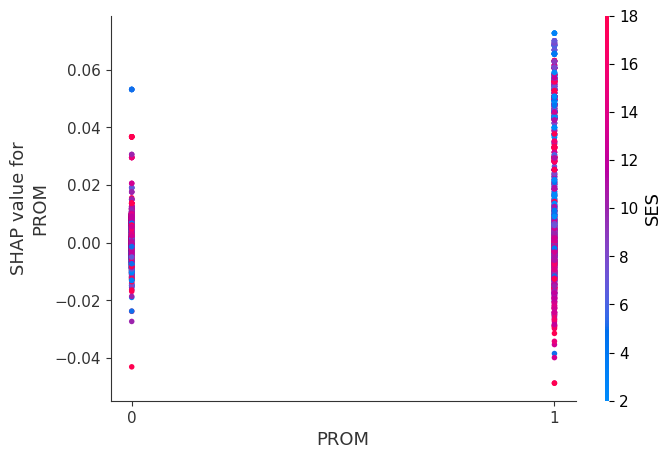

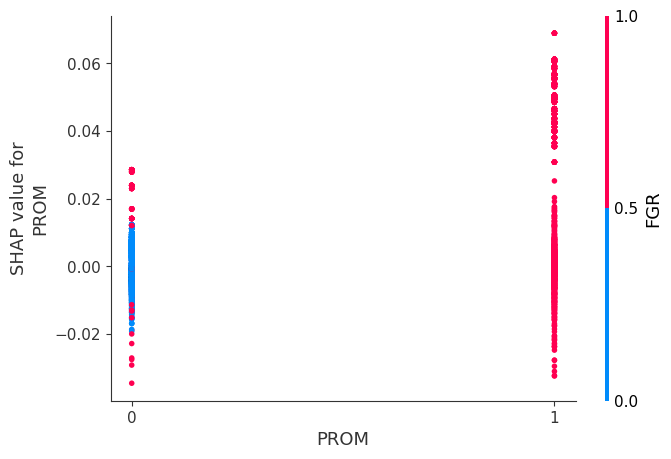


1. **Chorioamnionitis**

**a) Total b) Male c) Female**


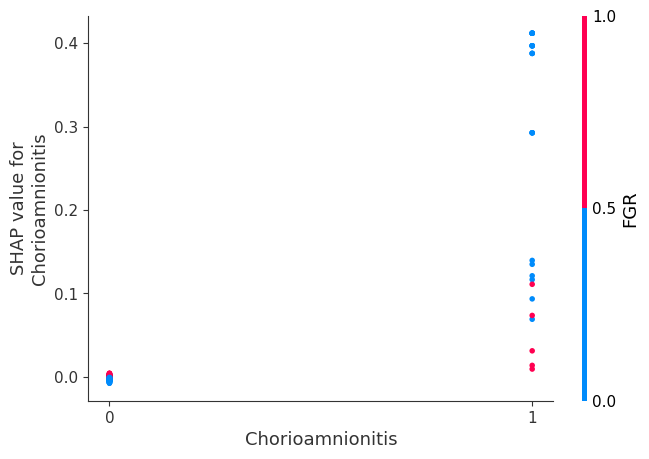

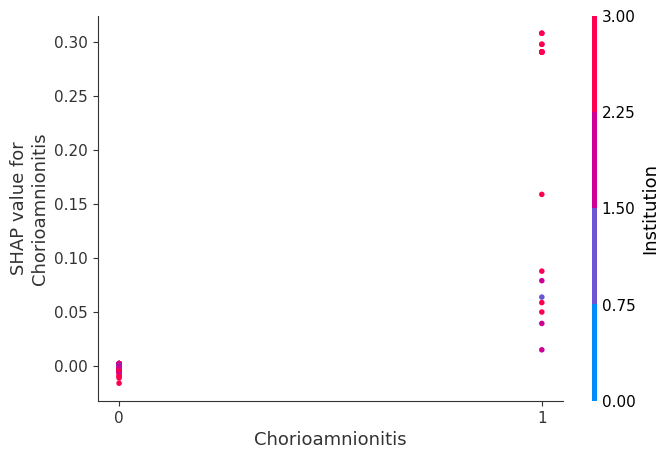

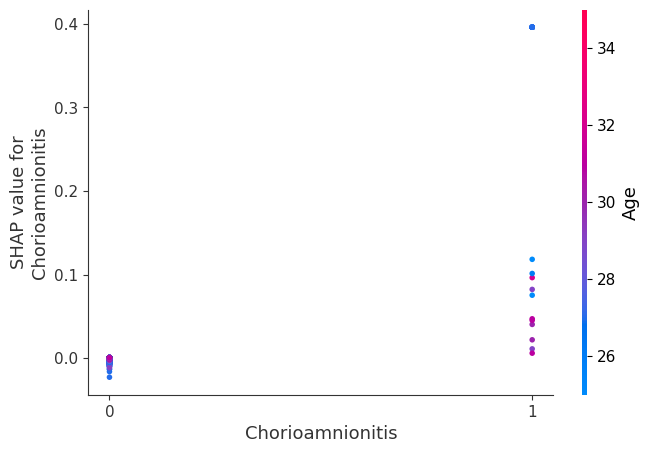


**Supplementary Figure 2. Shapley Addictive Explanations interaction plot**

**a) Total**


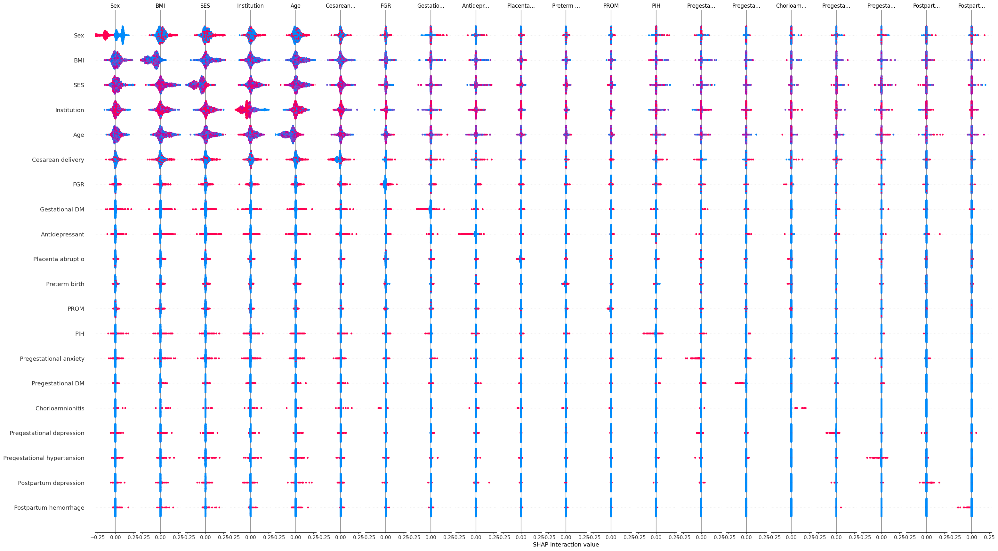


**b) Male**


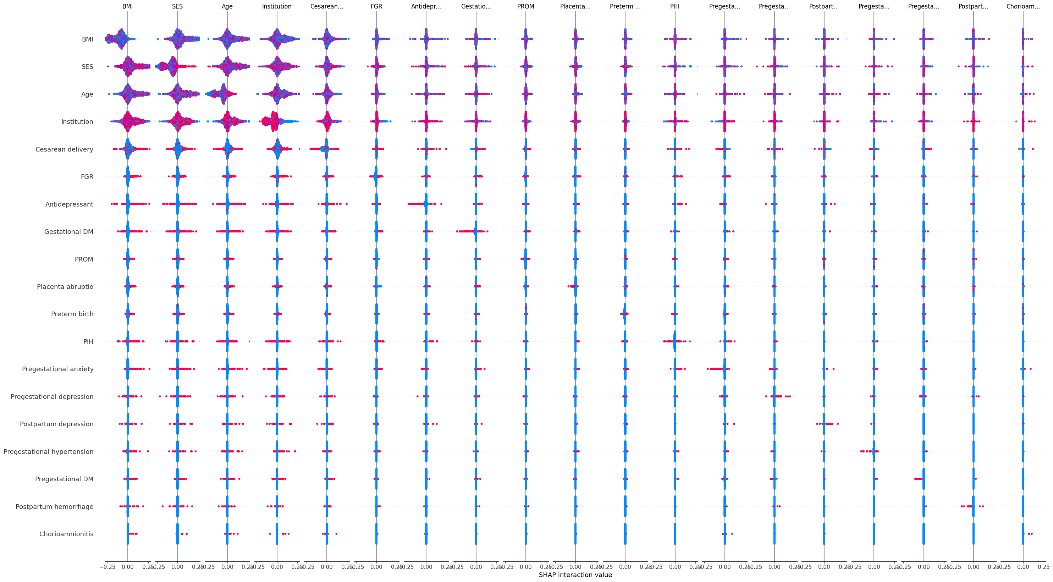


**c) Female**

**
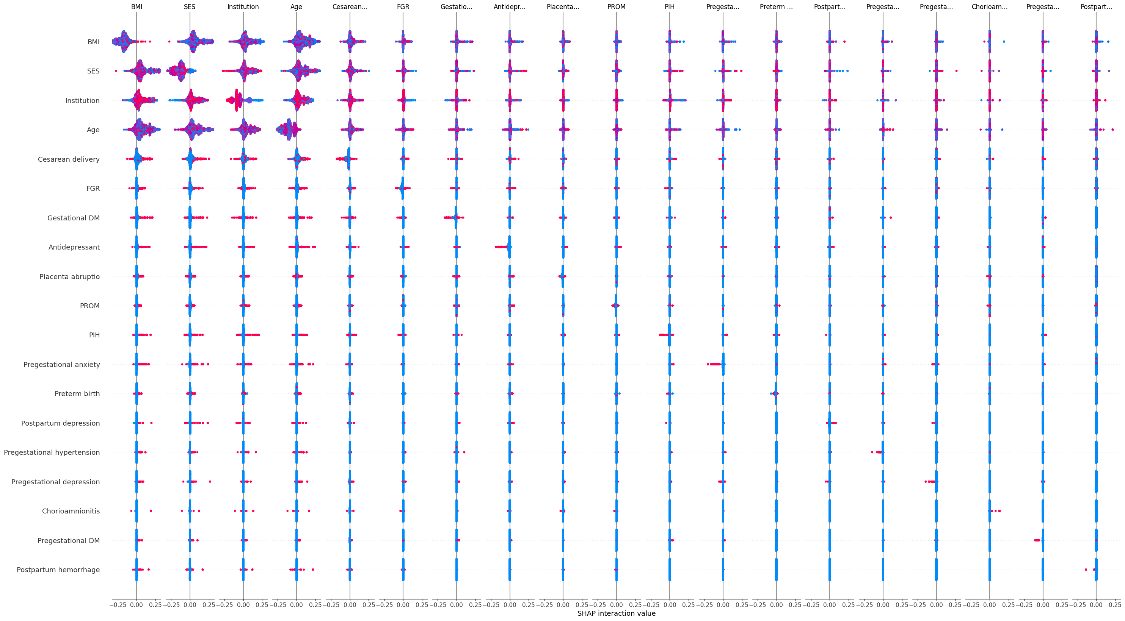
**
